# Chemical Composition and Pharmacological Effects of Geopropolis Produced by *Melipona quadrifasciata anthidioides*

**DOI:** 10.1155/2017/8320804

**Published:** 2017-10-26

**Authors:** Cintia Miranda dos Santos, Jaqueline Ferreira Campos, Helder Freitas dos Santos, José Benedito Perrella Balestieri, Denise Brentan Silva, Kely de Picoli Souza, Carlos Alexandre Carollo, Leticia M. Estevinho, Edson Lucas dos Santos

**Affiliations:** ^1^Research Group on Biotechnology and Bioprospecting Applied to Metabolism (GEBBAM), Federal University of Grande Dourados, Rodovia Dourados Itahum, Km 12, 79804-970 Dourados, MS, Brazil; ^2^Laboratory of Natural Products and Mass Spectrometry, Federal University of Mato Grosso do Sul, Cidade Universitária, 79070-900 Campo Grande, MS, Brazil; ^3^Agricultural College of Bragança, Polytechnic Institute of Bragança, Campus Santa Apolónia, 5301-855 Bragança, Portugal; ^4^Molecular and Environmental Biology Centre (CBMA), Universidade do Minho, Campus de Gualtar, 4710 057 Braga, Portugal

## Abstract

Stingless bees produce geopropolis, which is popularly described for its medicinal properties, but for which few scientific studies have demonstrated pharmacological effects. The objective of this study was to investigate the chemical composition of the geopropolis of *Melipona quadrifasciata anthidioides* and to evaluate its antioxidant, antimutagenic, anti-inflammatory, and antimicrobial activities. The composition of the hydroethanolic extract of geopropolis (HEG) included di- and trigalloyl and phenylpropanyl heteroside derivatives, flavanones, diterpenes, and triterpenes. HEG showed antioxidant action via the direct capture of free radicals and by inhibiting the levels of oxidative hemolysis and malondialdehyde in human erythrocytes under oxidative stress. HEG also reduced the frequency of gene conversion and the number of mutant colonies of *S. cerevisiae*. The anti-inflammatory action of HEG was demonstrated by the inhibition of hyaluronidase enzyme activity. In addition, HEG induced cell death in all evaluated gram-positive bacteria, gram-negative bacteria, and yeasts, including clinical isolates with antimicrobial drug resistance. Collectively, these results demonstrate the potential of *M. q. anthidioides* geopropolis for the prevention and treatment of various diseases related to oxidative stress, mutagenesis, inflammatory processes, and microbial infections.

## 1. Introduction

Geopropolis is produced by stingless bees (Hymenoptera, Apidae, and Meliponinae) [1] from a mixture of wax, pollen, and the mandibular secretions of bees together with plant resins and the addition of soil, which characterizes and differentiates this material [2, 3]. Geopropolis is deposited in the hive to seal cracks, delimit the cavities where bees reside, and prevent excessive air entry [4].

Analyses of the chemical compositions of geopropolis samples produced by different species of bees have demonstrated the complexity of this natural product, which contains phenolic compounds such as benzophenones [5], phenolic acids, hydrolysable tannins, and flavonoids [1, 6, 7], in addition to terpenes and long-chain fatty acids [8, 9].

The compounds found in geopropolis are likely responsible for the biological activities that have been described in several studies, including antimicrobial [7, 10, 11], anti-inflammatory [2, 12, 13], antinociceptive [14], gastroprotective [15], antioxidant [1, 6, 16], antiproliferative [5, 11], antimutagenic [7], and cytotoxic [17, 18] activities.

Among stingless bee species, *Melipona quadrifasciata* Lepeletier, 1836, popularly known as mandaçaia, is found in much of the Brazilian territory and is subdivided into two subspecies, *M. quadrifasciata quadrifasciata* and *M. quadrifasciata anthidioides* [19, 20], which are well described in the literature with respect to development and genetic diversity [19, 21–23]. However, studies examining the chemical composition and therapeutic properties of the natural products produced by these subspecies, such as honey, propolis, and geopropolis, remain scarce.

Kujumgiev et al. [24] described the presence of aromatic acids and di- and triterpene in the propolis produced by *M. q. anthidioides* and revealed its antimicrobial action against *Staphylococcus aureus*, *Escherichia coli*, and *Candida albicans*. Velikova et al. [25] attributed the inhibition of *S. aureus* by the propolis of *M. q. anthidioides* to the presence of the diterpene kaurenoic acid. Recently, Bonamigo et al. [26] described the presence of several other compounds in the propolis of this bee subspecies, such as stigmasterol, taraxasterol, vanillic acid, caffeic acid, quercetin, luteolin, and apigenin, and demonstrated its antioxidant and cytotoxic action.

With respect to the geopropolis of this subspecies, only Bankova et al. [8] investigated its chemical composition, demonstrating the presence of compounds such as palmitic acid, oleic acid, benzoic acid, cinnamic acid, vanillin, and coniferaldehyde. In this context, the objective of this study was to determine the chemical composition of the hydroethanolic extract of the geopropolis produced by the stingless bee *M. q. anthidioides*, found in the state of Mato Grosso do Sul, Brazil, and to examine its antioxidant, antimutagenic, anti-inflammatory, and antimicrobial activities.

## 2. Materials and Methods

### 2.1. Collection of Geopropolis Samples

Samples of *M. q. anthidioides* bee geopropolis were collected at the geographic coordinates 22°13′12^″^S and 54°49′2^″^W in the state of Mato Grosso do Sul, which is in the Central-West region of Brazil. Samples were stored at −20°C until analysis.

### 2.2. Preparation of the Hydroethanolic Extract of Geopropolis

The hydroethanolic extract of geopropolis (HEG) was prepared from 80 g of geopropolis and 240 mL of 70% ethanol. The mixture was continuously stirred (165 rpm) for 24 h at room temperature before being filtered. The extract was then concentrated in a rotary evaporator (Gehaka, São Paulo, SP, Brazil) at 40°C and lyophilized to obtain a dried extract. The yield was 4.8%, and the material was stored in the dark at −20°C.

### 2.3. Determination of Phenolic Compounds and Flavonoids

The concentration of phenolic compounds in HEG was determined by the Folin-Ciocalteu colorimetric method [27]. To this end, 0.5 mL of extract (100 *μ*g/mL) was added to 2.5 mL of the Folin-Ciocalteu reagent and 2 mL of sodium carbonate solution (Na_2_CO_3_). The mixture was incubated for 2 h at room temperature in the dark before its absorbance was measured at 760 nm in a spectrophotometer (PG Instruments Limited, Leicestershire, UK). Gallic acid (0.4–11 *μ*g/mL) was used as the standard to produce the calibration curve. The average of three readings was used to determine the content of phenolic compounds, expressed as mg of gallic acid equivalents per gram of extract (mg GAE/g extract).

The concentration of flavonoids was determined using the method described by Liberio et al. [2] with minor modifications. Specifically, 0.5 mL of extract (100 *μ*g/mL) was added to 4.5 mL of methanolic solution of 2% aluminum chloride hexahydrate (AlCl_3_·6H_2_O). The mixture was incubated for 30 min at room temperature in the dark before its absorbance was measured at 415 nm in a spectrophotometer (PG Instruments Limited, Leicestershire, UK). Quercetin (0.4–11 *μ*g/mL) was used as the standard to produce the calibration curve. The mean of three readings was used to determine the flavonoid content, expressed as mg of quercetin equivalent per gram of extract (mg QE/g extract).

### 2.4. Analysis of HEG by High-Performance Liquid Chromatography Coupled to a Diode Array Detector and Tandem Mass Spectrometry (HPLC-DAD-MS/MS)

Five microliters of HEG (1 mg/mL) was injected into an LC-20AD ultrafast liquid chromatograph (UFLC) (Shimadzu) connected in line with a diode array detector (DAD) (240–800 nm) and a mass spectrometer with electrospray ionization (ESI) and a quadrupole time-of-flight (QTOF) analyzer (120–1200 Da; micrOTOF-Q III, Bruker Daltonics). It was equipped with a C-18 Kinetex column (150 mm × 2.2 mm inner diameter, 2.6 *μ*m) with an oven temperature of 50°C. The mobile phase consisted of deionized water (A) and acetonitrile (B), both containing 0.1% formic acid, with the following gradient: 0–8 min, 3% B; 8–30 min, 3–25% B; and 30–60 min, 25–80% B. The gradient was followed by washing and reconditioning of the column (8 min). The flow rate was 0.3 mL/min.

### 2.5. Antioxidant Activity Assays

#### 2.5.1. DPPH^•^ Free Radical Capture

The 2,2-diphenyl-1-picrylhydrazyl (DPPH) radical-scavenging activity of the geopropolis extract was evaluated according to the method described by D. Gupta and R. K. Gupta [28] with modifications. Specifically, 200 *μ*L of HEG solubilized in 80% ethanol (0.1–200 *μ*g/mL) was mixed with 1800 *μ*L of the DPPH^•^ solution (0.11 mM). The mixture was homogenized and incubated for 30 min at room temperature in the dark before its absorbance was measured at 517 nm in a spectrophotometer (PG Instruments Limited, Leicestershire, UK). Ascorbic acid and butylated hydroxytoluene (BHT) were used as reference antioxidants. As a negative control, 80% ethanol alone was incubated with DPPH^•^ solution. Three independent experiments were performed in duplicate. The percent inhibition relative to the negative control was calculated using the following:

(1) $$\begin{array}{c} {DPPH}^{•}\;inhibition\;\left(\%\right)=\frac{1-{Abs}_{sample}}{{Abs}_{control}}\times 100. \end{array}$$

#### 2.5.2. ABTS^•+^ Free Radical Scavenging

The antioxidant capacity of HEG was also evaluated by the method described by Re et al. [29], in which 2,2′-azinobis(3-ethylbenzthiazoline-6-sulfonic acid) (ABTS) free radical-scavenging activity is examined. The ABTS^•+^ radical was formed by mixing 5 mL of the ABTS solution (7 mM) with 88 *μ*L of potassium persulfate solution (140 mM). The mixture was incubated for 12–16 h at room temperature in the dark before the ABTS^•+^ radical was diluted in absolute ethanol until it reached an absorbance of 0.70 ± 0.05 at 734 nm in a spectrophotometer (PG Instruments Limited, Leicestershire, UK). Next, 20 *μ*L of HEG solubilized in 80% ethanol (0.1–200 *μ*g/mL) was mixed with 1980 *μ*L of the ABTS^•+^ radical. The mixture was incubated for 6 min, and its absorbance was then measured at 734 nm. Ascorbic acid and BHT were used as positive controls. As a negative control, 80% ethanol alone was incubated with the ABTS^•+^ radical. Two independent experiments were performed in triplicate. The percent inhibition of the ABTS^•+^ radical relative to the negative control was calculated using the following:

(2) $$\begin{array}{c} {ABTS}^{•+}\;inhibition\;\left(\%\right)=\frac{{Abs}_{control}-{Abs}_{sample}}{{Abs}_{control}}\times 100. \end{array}$$

#### 2.5.3. Inhibition of Oxidative Hemolysis and Lipid Peroxidation in Human Erythrocytes

The procedures performed were approved by the Ethics Committee of the University Center of Grande Dourados (Centro Universitário da Grande Dourados (UNIGRAN)), Brazil (CEP number 123/12). The assays were performed as described by Campos et al. [30].

*(1) Preparation of Erythrocyte Suspensions*. For the hemolysis inhibition and malondialdehyde assays, 20 mL of peripheral blood was collected from healthy donors and packed in a tube containing sodium citrate. The blood was centrifuged at 2000 rpm for 10 min, and the plasma and leukocyte layers were removed. The erythrocytes were washed with 0.9% sodium chloride (NaCl), and a 10% erythrocyte suspension in 0.9% NaCl was further diluted to yield a final erythrocyte concentration of 2.5% for the experiments.

*(2) Hemolytic Activity and Inhibition of Oxidative Hemolysis*. To investigate whether HEG promotes hemolysis in erythrocytes, erythrocyte samples were preincubated at 37°C for 30 min in the presence of different concentrations of HEG (5–75 *μ*g/mL); 0.9% NaCl solution was then added, and the mixture was incubated for 240 min with periodic homogenization. The ability of HEG to inhibit oxidative hemolysis was assessed after preincubation of the extract (5–75 *μ*g/mL) with the erythrocytes at 37°C for 30 min; the oxidizing agent 2,2′-azobis(2-amidinopropane)dihydrochloride (AAPH; 50 mM) was then added, and the mixture was incubated for 240 min with periodic homogenization. Protection against hemolysis was evaluated after 120, 180, and 240 min of incubation. Hemolysis was assayed by centrifuging the samples at 1500 rpm for 10 min and then reading the absorbance of the supernatant at 540 nm in a spectrophotometer (PG Instruments Limited, Leicestershire, UK). The ascorbic acid control was maintained under the same conditions in both assays. As a solvent control, the erythrocytes were incubated with ethanol at a final concentration of 1%. Three independent experiments were conducted in duplicate. The percentage of hemolysis was calculated using the following formula, where *A* is the absorbance of the sample and *B* is the total hemolysis (erythrocytes incubated with distilled water).

(3) $$\begin{array}{c} Inhibition\;of\;hemolysis\;\left(\%\right)=\frac{A}{B}\times 100. \end{array}$$

*(3) Quantification of Malondialdehyde (MDA)*. To examine the ability of HEG to protect erythrocytes against lipid peroxidation, the levels of MDA, a byproduct of lipid peroxidation, were quantified. HEG (5–75 *μ*g/mL) was preincubated with an erythrocyte suspension at 37°C for 30 min; the oxidizing agent AAPH (50 mM) was then added, and the mixture was incubated for 240 min with periodic homogenization. After this period, the samples were centrifuged at 1500 rpm for 10 min, and 500 *μ*L aliquots of the supernatant was transferred to tubes containing 1 mL of 10 nM thiobarbituric acid (TBA) solubilized in monosodium potassium phosphate buffer (75 mM) at pH 2.5. As an MDA standard, 500 *μ*L of 20 mM MDA solution was added to 1 mL of TBA. The samples were incubated at 96°C for 45 min before being cooled in an ice bath; 4 mL of n-butyl alcohol was then added, and the mixture was centrifuged at 3000 rpm for 5 min. The absorbance of the supernatant was read at 540 nm in a spectrophotometer (PG Instruments Limited, Leicestershire, UK). The ascorbic acid control was maintained under the same conditions. As a solvent control, the erythrocytes were incubated with ethanol at a final concentration of 1%. Three independent experiments were conducted in duplicate. MDA levels are expressed as nM/mL and were obtained using the following:

(4) $$\begin{array}{c} MDA={Abs}_{sample}\times \frac{20\times 220.32}{{Abs}_{MDA\;standard}}. \end{array}$$

### 2.6. Antimutagenic Activity

The antimutagenic activity of HEG was determined using *Saccharomyces cerevisiae* cells (D7 diploid strain of ATCC 201137) according to the method of Pascoal et al. [31]. Before each experiment, *S. cerevisiae* strains (*MAT*a/*MAT*a, *ade2-40*/*ade 2-119*, *trp 5-12*/*trp 5-27*, and *ILV 1-92*/*ILV 1-92*) were tested for the frequencies of spontaneous conversions at the tryptophan locus and revertants at the isoleucine locus. Cells from a culture with a low frequency of spontaneous gene conversion and a low back mutation (reversion of point mutation) frequency were grown in liquid medium at 28°C until they reached a stationary phase. *S. cerevisiae* cells were sedimented and resuspended in sterile potassium phosphate buffer (0.1 M; pH 7.4) to obtain a final concentration of 2 × 10^8^ cells/mL. As a mutagenic compound, ethyl methanesulfonate (EMS; 1 mg/mL) was added and incubated with the cell suspension, the potassium phosphate buffer, and HEG at final concentrations of 1.5 and 3.0 mg/mL. The mixture was incubated with stirring for 2 h at 37°C. The cells were then plated on complete and selective media to evaluate survival, tryptophan convertants, and isoleucine revertants. The experiments were performed in triplicate.

### 2.7. Anti-Inflammatory Activity Assessment by Hyaluronidase Enzyme Inhibition

The anti-inflammatory potential of HEG was evaluated indirectly by examining its inhibition of hyaluronidase enzyme activity according to the method described by Silva et al. [32]. The reaction mixture consisted of 50 *μ*L of HEG and 50 *μ*L (350 units) of hyaluronidase (type IV-S; bovine testes, Sigma, St. Louis, MO, USA), which was incubated at 37°C for 20 min. Next, 1.2 *μ*L of calcium chloride (2.5 × 10^−3^ M) was added, and the mixture was incubated at 37°C for 20 min to activate the enzyme. As a substrate, 500 *μ*L of hyaluronic acid sodium salt (0.1 M) was added. The mixture was incubated at 37°C for 40 min, 100 *μ*L of potassium tetraborate (0.8 M) was then added, and the mixture was incubated at 100°C for 3 min. After cooling the solution, 3 mL of p-dimethylaminobenzaldehyde was added, and the mixture was incubated at 37°C for 20 min. Its absorbance was then measured at 585 nm in a spectrophotometer (PG Instruments Limited, Leicestershire, UK). Distilled water was used as a control. The experiments were performed in triplicate. The percent inhibition of enzyme activity relative to the control was calculated using the following:

(5) $$\begin{array}{c} Inhibition\;of\;hyaluronidase\;activity\;\left(\%\right)=\frac{{Abs}_{control}-{Abs}_{sample}}{{Abs}_{control}}\times 10. \end{array}$$

### 2.8. Antimicrobial Activity

The microorganisms used in this study are listed in Table 1. The clinical microorganisms were isolated from biological fluids collected at the Hospital Center and were identified at the Laboratory of Microbiology of the School of Higher Agricultural Education of Bragança (Escola Superior Agrária de Bragança (ESA)), and reference strains were obtained from the American Type Culture Collection (ATCC) (LGC Standards SLU, Barcelona, Spain).

Prior to experimental use, the isolates were maintained in Muller-Hinton medium containing 20% glycerol at −70°C. The inoculum for the assays was prepared by dilution of a cell mass in 0.85% NaCl solution to 0.5 on the McFarland scale and confirmed by spectrophotometry at 580 and 640 nm for bacteria and yeast, respectively. For antimicrobial assays, microorganism suspensions were diluted to 10^4^ colony-forming units (CFU)/mL according to the method described by Silva et al. [32]. Nutrient broth (NB) or yeast extract-peptone-dextrose (YPD) was used in microplates (96 wells). The HEG was diluted in dimethyl sulfoxide (DMSO) and transferred to the first well of the plate; serial dilutions were then performed. The inoculum was added to all wells, and the plates were incubated at 37°C for 24 h (bacteria) or at 25°C for 48 h (yeast). As positive controls, the antibiotic gentamicin and the antifungal amphotericin B were used. In addition, media controls were conducted with and without inocula, and DMSO alone was used as a solvent control in inoculated medium. Antimicrobial activity was detected by the addition of 20 *μ*L of 2,3,5-triphenyl-2H-tetrazolium chloride (TTC) solution (5 mg/mL). The minimal inhibitory concentration (MIC) was defined as the lowest concentration of HEG that inhibited visible growth of the microorganisms as indicated by TTC staining of living cells.

To determine the minimum bactericidal concentration (MBC) and minimum fungicidal concentration (MFC), 20 *μ*L samples was collected from the last well where growth was observed and from each well that did not show a change in staining, and the samples were plated on NB or YPD and incubated at 37°C for 24 h (bacteria) or at 25°C for 48 h (yeast). The MBC or MFC was defined as the lowest concentration that did not result in growth (<10 CFU/plate) after cultivation. The results are expressed as mg/mL, and the experiments were performed in triplicate.

### 2.9. Statistical Analyses

The data are expressed as the mean ± standard error of the mean (SEM) and were evaluated by ANOVA followed by the Dunnett's test using GraphPad Prism Software version 5.0 (GraphPad Software Inc., San Diego, CA, USA). The results were considered significant when *P* < 0.05.

## 3. Results

### 3.1. Chemical Composition

The total concentrations of phenolic compounds and flavonoids present in HEG were 118.7 ± 2.8 mg GAE/g extract and 25.4 ± 2.8 mg QE/g extract, respectively.

**Figure 1:**
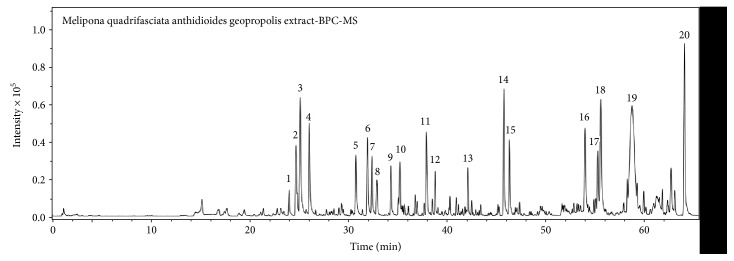
Base peak chromatogram of *M. q. anthidioides* geopropolis extract. Peaks 1 to 20 are identified in Table 1.

Peaks 1, 2, 4, 5, 6, 7, 9, 11, and 12 showed similar UV spectra and MS/MS fragments. The UV absorbance of the compounds was centered at approximately 300 nm, and the observed variations were compatible with the presence of different moieties (Figure 1, Table 2). All of these compounds exhibited a central hexose with two or three galloyl, cinnamoyl, or coumaroyl groups. Certain key fragments supported putative identities of the peaks, such as *m*/*z* 169 (C_7_H_5_O_5_)^−^, related to gallic acid, observed in compounds 2, 3, 4, 5, 6, 9, and 12; *m*/*z* 313 (C_13_H_13_O_9_)^−^, related to a galloyl-hexoside fragment, detected in compounds 1, 2, 4, 6, 9, and 12; and *m*/*z* 465 (C_20_H_17_O_13_)^−^, related to a digalloyl-hexoside moiety, observed in compounds 4 and 6. Other fragments yielded less information, as in the case of *m*/*z* 265 (C_13_H_13_O_6_)^−^, *m*/*z* 235 (C_12_H_11_O_5_)^−^, and *m*/*z* 205 (C_11_H_9_O_4_)^−^ in compound 2 or *m*/*z* 145 (C_9_H_5_O_2_)^−^ in compounds 7 and 8; these fragments are likely the result of substitutions at specific positions of the hexose. Unfortunately, the determination of these sites was not possible due to a lack of information in the literature or a limited access to standards. Thus, compounds were identified as coumaroyl-galloyl-hexoside derivatives (1 and 2), digalloyl-coumaroyl-hexoside (4), cinnamoyl-galloyl-hexoside (5), digalloyl-cinnamoyl-hexoside (6), dicoumaroyl-hexoside (7), dicoumaroyl-galloyl-hexoside (9), cinnamoyl-coumaroyl-hexoside (11), and cinnamoyl-coumaroyl-galloyl-hexoside (12); compounds of this class were also isolated from the geopropolis of *M. subnitida* [1].

The HEG also contained three observed flavanones (peaks 3, 8, and 10). They were characterized based on their UV spectra, high-resolution mass, and fragments such as aromadendrin, naringenin, and methyl aromadendrin, which were previously reported in the geopropolis of *Melipona* ssp. [1, 16].

The final characterized peaks were detected at the end of the chromatogram (peaks 14, 15, 16, 17, 18, 19, and 20; Figure 1), implying that they were apolar compounds. These peaks did not absorb in the UV-monitored range, and their molecular formulae suggest diterpene (peaks 14 and 15) and triterpene (peaks 16, 17, and 18) derivatives. Although these chemical classes are very common in propolis and geopropolis [24, 25], no more information could be obtained due to a lack of fragmentation and the large number of possible skeletons.

Compounds 19 (C_24_H_38_O_3_) and 20 (C_24_H_36_O_3_) showed similar formulae, differing by only two hydrogens; both showed only one fragment that was related to CO_2_ loss, suggesting the presence of carboxylic acid in the structure. However, no compatible plant metabolites were found in the literature.

### 3.2. Antioxidant Activity

#### 3.2.1. DPPH^•^ and ABTS^•+^ Free Radical Scavenging

**Figure 2:**
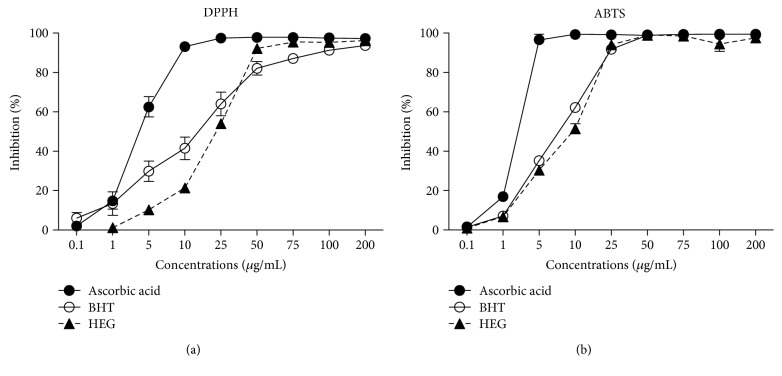
Antioxidant activity, as shown by scavenging of the free radicals (a) DPPH^•^ and (b) ABTS^•+^ by the ascorbic acid and BHT controls and by the *M. q. anthidioides* geopropolis extract (0.1–200 *μ*g/mL).

The HEG displayed relevant antioxidant action in the direct scavenging of the free radicals DPPH^•^ and ABTS^•+^. In the DPPH^•^ assay, HEG inhibited 50% of the free radicals (IC_50_) at a concentration of 28.9 ± 1.3 *μ*g/mL, an activity 1.7 times lower than that of the antioxidant BHT (IC_50_ = 16.9 ± 5.2 *μ*g/mL) (Figure 2(a)). In the ABTS^•+^ assay, the HEG showed an IC_50_ of 9.5 ± 0.8 *μ*g/mL, similar to the synthetic antioxidant BHT (IC_50_ = 8.1 ± 0.7 *μ*g/mL); however, HEG showed five times less activity than the standard antioxidant ascorbic acid (IC_50_ = 1.8 ± 0.05 *μ*g/mL) (Figure 2(b)).

#### 3.2.2. Hemolytic Activity and Inhibition of Oxidative Hemolysis

**Figure 3:**
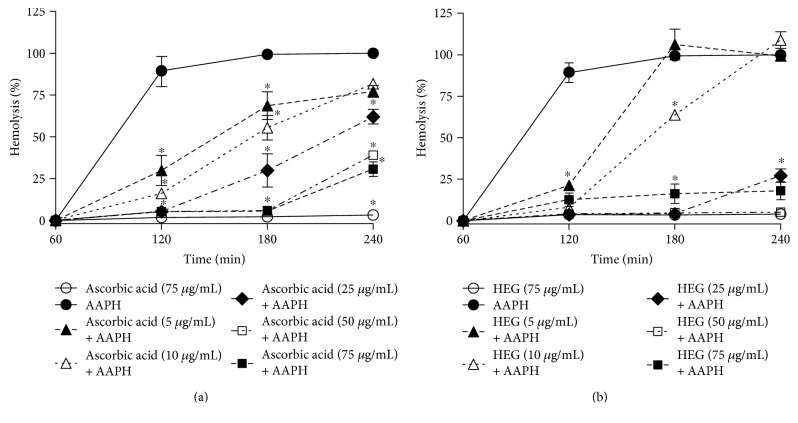
Inhibition of oxidative hemolysis in human erythrocytes incubated with (a) ascorbic acid and (b) HEG (5–125 *μ*g/mL) in the presence of the oxidizing agent AAPH for 240 min. ^∗^Statistically significant results (*P* < 0.05) compared to those of the AAPH control group at the same time point.

Human erythrocytes incubated with the highest concentrations (75 *μ*g/mL) of ascorbic acid (Figure 3(a)) or HEG (Figure 3(b)) did not show hemolysis, indicating that these compounds did not promote changes in this cellular model.

In the presence of the oxidizing agent AAPH, HEG protected the erythrocytes against oxidative hemolysis throughout the experimental period (Figure 3(b)). After 240 min, HEG inhibited 80.9 ± 5.6% of oxidative hemolysis at 75 *μ*g/mL, demonstrating superior antihemolytic action relative to that of ascorbic acid, which protected 67.6 ± 4.5% of erythrocytes at the same concentration (Figure 3(a)).

#### 3.2.3. Inhibition of MDA Production

**Figure 4:**
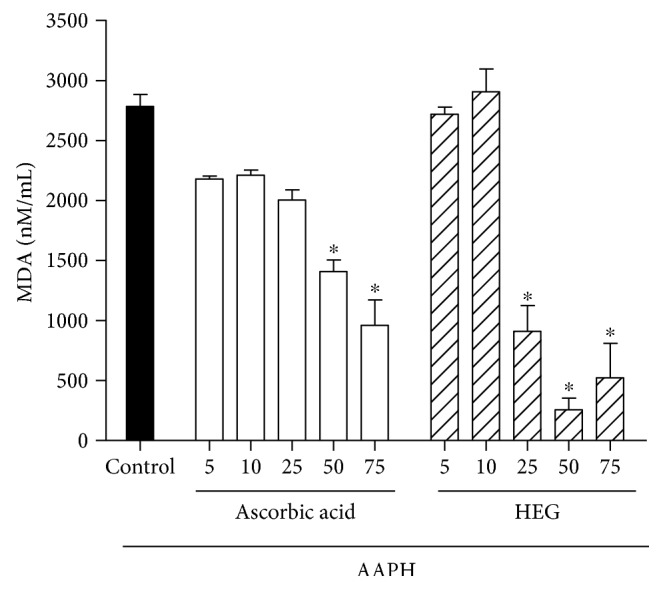
Malondialdehyde concentration (MDA) in nM/mL after incubation of erythrocytes for 240 min with ascorbic acid or HEG (5–125 *μ*g/mL) in the presence of the oxidizing agent AAPH. ^∗^Statistically significant results (*P* < 0.05) compared to those of the AAPH control group.

The ability of the extract to inhibit lipid peroxidation was assessed by measuring MDA levels. After 240 min, 75 *μ*g/mL HEG was able to reduce MDA levels by 81.2 ± 10%, whereas the standard antioxidant ascorbic acid inhibited 65.5 ± 7.7% of MDA production at the same concentration (Figure 4).

### 3.3. Antimutagenic Activity

**Figure 5:**
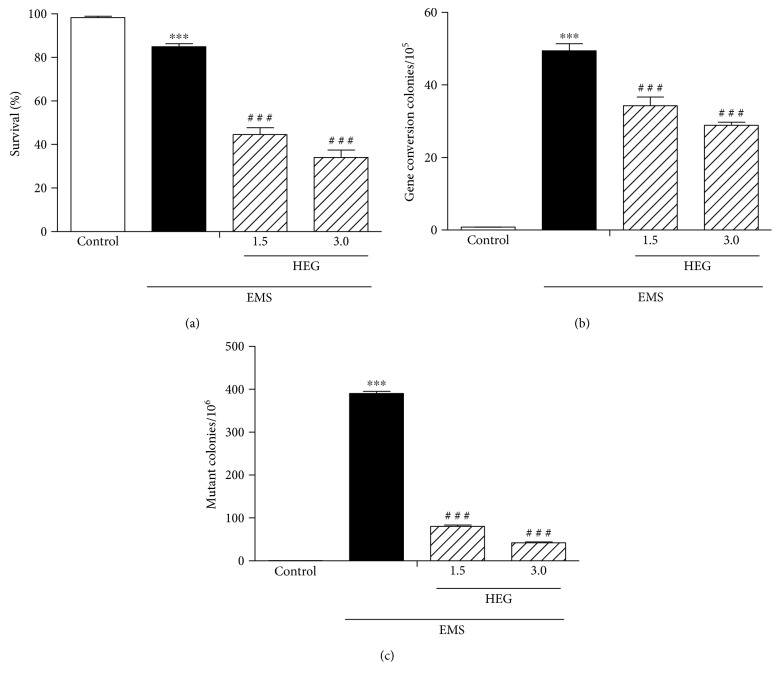
*S. cerevisiae* yeast cells (D7 diploid strain of ATCC 201137) incubated with HEG (1.5 and 3.0 mg/mL) and the mutagen EMS. (a) Survival percentage, (b) gene conversion, and (c) mutant colonies are shown. ^∗∗∗^*P* < 0.0001 compared to the control. ^#^^#^^#^*P* < 0.0001 compared to the EMS control.

The HEG of *M. q. anthidioides* showed an antimutagenic effect on *S. cerevisiae* cells incubated with the mutagen EMS. HEG reduced the survival of *S. cerevisiae* D7 by approximately 50% (Figure 5(a)), possibly because of its fungicidal action, and showed an antimutagenic effect by inhibiting the DNA damage promoted by EMS. The HEG reduced gene conversion frequencies by 30.7 ± 4.8% and 41.5 ± 1.7% at concentrations of 1.5 and 3.0 mg/mL, respectively (Figure 5(b)). In addition, HEG reduced the number of mutant colonies by 79.4 ± 0.8% at 1.5 mg/mL and by 89.3 ± 0.5% at 3.0 mg/mL (Figure 5(c)).

### 3.4. Anti-Inflammatory Activity

**Figure 6:**
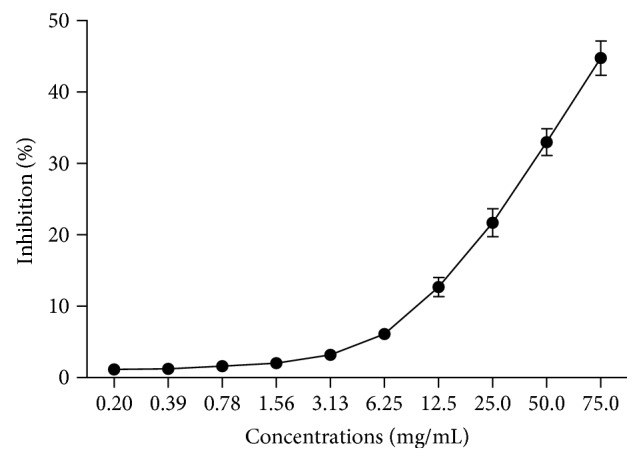
Inhibition of the inflammatory enzyme hyaluronidase by HEG (0.2–75 mg/mL).

The anti-inflammatory activity of HEG was evaluated by testing its ability to inhibit hyaluronidase enzyme activity in the presence of its substrate, hyaluronic acid sodium salt. The extract showed a concentration-dependent profile, inhibiting 44.7 ± 2.4% of hyaluronidase activity at 75 mg/mL (Figure 6).

### 3.5. Antimicrobial Activity

HEG showed antimicrobial activity against all of the tested microorganisms, including antimicrobial drug-resistant strains (Table 3). Gram-positive bacteria were more sensitive to HEG than were gram-negative species. The most sensitive microorganism was *Staphylococcus aureus* ATCC 6538, which showed an MIC of 5.16 ± 0.22 mg/mL and an MBC of 7.33 ± 0.16 mg/mL. The strain with the greatest resistance was the gram-negative bacterium *Pseudomonas aeruginosa* ESA 23, which is resistant to imipenem and originated from gingival exudate in a hospital setting; it showed an MIC of 12.75 ± 0.28 mg/mL and an MBC of 16.41 ± 0.36 mg/mL.

HEG also exhibited antifungal activity against all of the tested yeasts, including reference strains and those of hospital origin. The strain most sensitive to the action of HEG was *Cryptococcus neoformans* ATCC 32264, and the most resistant strain was *Candida albicans* ESA 97, which is resistant to amphotericin B and is of hospital origin.

## 4. Discussion

The literature contains few scientific studies related to the chemical composition and pharmacological properties of geopropolis, an apicultural product with great pharmacological potential. In folk medicine, it is prescribed for the treatment of digestive, respiratory, and visual problems [33] and as an antiseptic [18]. These activities are related to the chemical composition of geopropolis, which is dependent on the local flora, the producing bee species, and the type of soil found in the region of geopropolis production [1, 3].

Phenolic heterosides, flavanones, and terpenoids were observed in the HEG; these compounds are frequently detected in *Apis mellifera* propolis [34, 35] but have not often been described for *Melipona* geopropolis. An important finding was the presence of di- and trigalloyl and phenylpropanyl heteroside derivatives, which are rarely reported in the literature. Despite the paucity of reports, this class of compounds occurs in the kino of *Eucalyptus* spp. [36, 37], a common plant in the region where the geopropolis was collected. This observation may provide a clue about the source of raw material for the production of geopropolis by *Melipona* spp.

Other information about the origin of geopropolis came from a study performed by Sawaya et al. [38] with the stingless bee *Tetragonisca angustula* that reported compounds 19 and 20 in the propolis of this species and in the flowers and leaves of *Schinus terebinthifolius*. We analyzed the methanolic extract of the leaves of *S. terebinthifolius* from the same region where the geopropolis was collected and confirmed the presence of compound 20 (see Supplementary Material available online at https://doi.org/10.1155/2017/8320804). We conclude that *M. q. anthidioides* uses vegetal material from *S. terebinthifolius* to produce geopropolis. Flavanones and terpenes, the other two classes of identified compounds, are also normally present in apicultural plants [39–41], suggesting that *Melipona* spp. can use the same plants that *Apis* spp. use to produce propolis.

The phenolic compounds are described as important antioxidant agents [7, 26, 30], and the content present in the HEG was approximately two times more than that present in geopropolis from other species of bees, as *Melipona subnitida* [1] and *Melipona fasciculata* [6]. Among the phenolic compounds identified in the HEG, the flavonoids methyl aromadendrin [42] and naringenin [43] are described as having antioxidant activities. In this study, HEG was able to scavenge the free radicals DPPH^•^ and ABTS^•+^, showing results similar to those presented by the geopropolis of *M. subnitida* [1] and higher compared to those by the geopropolis of the species *Melipona interrupta* and *Melipona seminigra* [16].

Moreover, HEG protected human erythrocytes against damage generated by the oxidizing agent AAPH, resulting in lower levels of both oxidative hemolysis and malondialdehyde (as a marker of lipid peroxidation). Flavonoids inhibit peroxyl radicals via the donation of hydrogen atoms, a process enabled by the presence of a dihydroxylated B ring in their structures [44]. Peroxyl radicals are involved in the lipid peroxidation process, which creates a number of degradation products, such as the aldehydes 4-hydroxy-2-nonenal, 2-propenal, and malondialdehyde [45]. Naringenin, one of the flavonoids identified in HEG, has been described as modulating the activity of the enzymes superoxide dismutase, glutathione peroxidase, and catalase, in addition to protecting the cell membrane against the lipid peroxidation process by inhibiting the production of malondialdehyde and increasing the content of thiol-SH groups [43]. In turn, diterpenes promote the scavenging of free radicals, which results in the inhibition of lipid peroxidation [46, 47].

Lipid peroxidation resulting from elevated cellular oxidative stress is capable of promoting damage to proteins and nucleic acids, which may constitute the first steps of mutagenesis and carcinogenesis [48, 49]. With this perspective, after confirming the antioxidant activity of HEG from *M. q. anthidioides*, its antimutagenic properties were investigated. The HEG minimized the damage induced by the EMS alkylating agent, which induces random mutations in DNA by nucleotide substitution [31]. The antimutagenic activity of the HEG was similar to that of the geopropolis extract of *Melipona orbignyi*, regarding the reduction of mutant colonies; however, it was more effective against the gene conversion [7].

Living organisms have mutation repair mechanisms; however, they are often unable to correct all of the changes that occur, leaving the cell vulnerable to the development of problems related to DNA alterations [50]. Certain changes in the genetic material of cells are related to the development of cancer [51, 52]. Thus, products with antimutagenic activities can aid in preventing various types of cancers.

In addition to its antioxidant and antimutagenic activities, there are reports in the literature about the anti-inflammatory action of geopropolis, both *in vitro* and *in vivo*, via modulation of the main inflammatory mediators [12, 14]. In this study, HEG showed anti-inflammatory action in its inhibition of hyaluronic acid degradation by the hyaluronidase enzyme. Hyaluronic acid is a polysaccharide found primarily in the extracellular and pericellular matrix as a component of soft connective tissues [53], where it has an important role in tissue renewal [32]. The degradation of hyaluronic acid by hyaluronidase results in tissue permeability [54], bone loss, inflammation, and pain [31, 54].

The inhibition of hyaluronidase activity by HEG may be related to the presence of flavonoids in its composition, as they have been described as reducing hyaluronidase activity by binding to the enzyme and promoting structural alterations by means of electrostatic forces and hydrophobic interactions [55]. In addition, the flavonoids identified in HEG, naringenin and 7-O-methylaromadendrin, reportedly inhibit the activity of the cyclooxygenase 1 enzyme, and aromadendrin inhibits the activity of xanthine oxidase [56]; these enzymes are directly related to inflammatory processes.

Another important pharmacological activity exhibited by HEG was its antimicrobial action against both reference and clinical strains. HEG promoted the death of all of the tested gram-positive bacteria, gram-negative bacteria, and yeasts. Previous studies by Kujumgiev et al. [24] and Velikova et al. [25] described the antimicrobial potential of propolis from *M. q. anthidioides*; however, this is the first report to show that the geopropolis of this bee subspecies can combat pathogenic microorganisms.

The identification of new compounds with antimicrobial action has aroused the interest of the pharmaceutical industry, especially because many strains of microorganisms have become resistant to currently available antimicrobial drugs, thereby resulting in high morbidity and mortality rates, especially among nosocomial infections [57, 58]. Among the main mechanisms of resistance are the elimination of antibiotics by efflux pumps present in the cell wall of microorganisms [59, 60] and the degradation of antibiotics by specific enzymes [61].

Natural products represent a good source for the discovery of bioactive compounds with high antimicrobial activity, especially considering the complexity of the molecules present in these bioproducts [62]. The antimicrobial properties of geopropolis from other bee species have been described in other studies with *Melipona fasciculata* [2, 63], *Melipona orbignyi* [7], *Melipona scutellaris* [10, 11], and *Melipona mondury* [64], as well as the subspecies *Melipona compressipes fasciculate* [65].

Among the chemical compounds described as responsible for the antimicrobial activity of natural products are the flavonoids [66, 67]. In bacteria, they are capable of inhibiting DNA gyrase [68], damaging cell membranes by reducing their fluidity [69] and decreasing microbial energy metabolism [66]; these mechanisms are responsible for their antibacterial effects. In addition to these, diterpenes are also described as having antimicrobial activity, as they easily penetrate the cell membranes of microorganisms and create pores that result in the loss of intracellular contents [44].

In this context, this study is the first to report the chemical composition and pharmacological activities of the geopropolis extract of *M. q. anthidioides*, a natural product of apicultural origin that has great potential to be used in the prevention and treatment of several diseases related to oxidative stress, mutagenesis, inflammatory processes, and microbial infections.

## Supplementary Material

Supplementary Material. Base peak chromatogram of methanolic extract of the leaves of *Schinus terebinthifolius* in negative ionization mode.

## Figures and Tables

**Table 1:** Microorganisms used in the present study to test the antimicrobial activity of HEG from *M. q. anthidioides.*

Microorganism	Reference	Origin
*Staphylococcus aureus*	ATCC 6538™	Reference culture
Methicillin-resistant *S. aureus*	ESA 175	Pus
Methicillin-resistant *S. aureus*	ESA 159	Expectoration
*Enterococcus faecalis*	ATCC 43300™	Reference culture
Vancomycin-resistant *E. faecalis*	ESA 201	Urine
Vancomycin-resistant *E. faecalis*	ESA 361	Rectal swabs
*Escherichia coli*	ATCC 29998™	Reference culture
Cephalosporin-resistant *E. coli*	ESA 37	Urine
Cephalosporin-resistant *E. coli*	ESA 54	Hemoculture
*Pseudomonas aeruginosa*	ATCC 15442™	Reference culture
Imipenem-resistant *P. aeruginosa*	ESA 22	Expectoration
Imipenem-resistant *P. aeruginosa*	ESA 23	Gingival exudates
*Cryptococcus neoformans*	ATCC 32264	Reference culture
Amphotericin B-resistant *C. neoformans*	ESA 211	Blood
Amphotericin B-resistant *C. neoformans*	ESA 105	Skin biopsy
*Candida albicans*	ATCC 10231™	Reference culture
Amphotericin B-resistant *C. albicans*	ESA 100	Feces
Amphotericin B-resistant *C. albicans*	ESA 97	Urine

**Table 2:** Compounds identified from *Melipona quadrifasciata anthidioides* geopropolis extract by HPLC-DAD-ESI-qTOF-MS/MS.

Peak	RT (min)	UV (nm)	MS (*m*/*z*)	Molecular formula	Error (ppm)	MS/MS (*m*/*z*)	Identification
1	24	289 and 309	477.1053	C_22_H_22_O_12_	2.9	313 (C_13_H_13_O_9_)^−^, 271 (C_11_H_11_O_8_)^−^, 169 (C_7_H_5_O_5_)^−^	Coumaroyl-galloyl-hexoside
2	24.7	289 and 309	477.1040	C_22_H_22_O_12_	0.5	313 (C_13_H_13_O_9_)^−^, 265 (C_13_H_13_O_6_)^−^, 235 (C_12_H_11_O_5_)^−^, 205 (C_11_H_9_O_4_)^−^, 169 (C_7_H_5_O_5_)^−^	Coumaroyl-galloyl-hexoside
3	25.1	289	287.0565	C_15_H_12_O_6_	1.6	259 (C_14_H_11_O_5_)^−^, 177 (C_10_H_9_O_3_)^−^	Aromadendrin
4	26	286 and 308	629.1166	C_29_H_26_O_16_	3.1	465 (C_20_H_17_O_13_)^−^, 459 (C_22_H_19_O_11_)^−^, 313 (C_13_H_13_O_9_)^−^, 271 (C_11_H_11_O_8_)^−^, 169 (C_7_H_5_O_5_)^−^	Digalloyl-coumaroyl-hexoside
5	30.8	280	461.1113	C_22_H_22_O_11_	3.7	211 (C_9_H_7_O_6_)^−^, 169 (C_7_H_5_O_5_)^−^, 161 (C_10_H_9_O_2_)^−^	Cinnamoyl-galloyl-hexoside
6	31.9	280	613.1216	C_29_H_26_O_15_	2.2	465 (C_20_H_17_O_13_)^−^, 313 (C_13_H_13_O_9_)^−^, 271 (C_11_H_11_O_8_)^−^, 211 (C_9_H_7_O_6_)^−^, 169 (C_7_H_5_O_5_)^−^	Digalloyl-cinnamoyl-hexoside
7	32.4	299 and 311	471.1324	C_24_H_24_O_10_	4.3	325 (C_15_H_17_O_8_)^−^, 307 (C_15_H_15_O_7_)^−^, 265 (C_13_H_13_O_6_)^−^, 163 (C_9_H_7_O_3_)^−^, 145 (C_9_H_5_O_2_)^−^	Dicoumaroyl-hexoside
8	32.9	284	271.0622	C_15_H_12_O_5_	3.2	—	Naringenin
9	34.3	290 and 311	623.1428	C_31_H_28_O_14_	2.2	459 (C_22_H_19_O_11_)^−^, 313 (C_13_H_13_O_9_)^−^, 271 (C_11_H_11_O_8_)^−^, 211 (C_9_H_7_O_6_)^−^, 169 (C_7_H_5_O_5_)^−^,163 (C_9_H_7_O_3_)^−^	Dicoumaroyl-galloyl-hexoside
10	35.2	290	301.0731	C_16_H_14_O_6_	2.8	273 (C_15_H_13_O_5_)^−^, 240 (C_14_H_8_O_4_)^−^, 179 (C_8_H_3_O_5_)^−^, 165 (C_8_H_5_O_4_)^−^	Methyl aromadendrin
11	37.9	285 and 310	455.1353	C_24_H_24_O_9_	1.2	163 (C_9_H_7_O_3_)^−^, 145 (C_9_H_5_O_2_)^−^	Cinnamoyl-coumaroyl-hexoside
12	38.8	285 and 310	607.1455	C_31_H_28_O_13_	2.7	461 (C_22_H_21_O_11_)^−^, 443 (C_22_H_19_O_10_)^−^, 313 (C_13_H_13_O_9_)^−^, 271 (C_11_H_11_O_8_)^−^, 211 (C_9_H_7_O_6_)^−^, 169 (C_7_H_5_O_5_)^−^	Cinnamoyl-coumaroyl-galloyl-hexoside
13	42.1	295	421.1290	C_24_H_22_O_7_	0.9	—	Unknown
14	45.7	—	319.2272	C_20_H_32_O_3_	3.4	—	Diterpene
15	46.3	—	319.2270	C_20_H_32_O_3_	0.9	—	Diterpene
16	54	—	471.3475	C_30_H_48_O_4_	0.8	—	Triterpene
17	55.3	—	471.3471	C_30_H_48_O_4_	3.3	—	Triterpene
18	55.5	—	469.3314	C_30_H_46_O_4_	3.0	—	Triterpene
19	58.7	—	373.2736	C_24_H_38_O_3_	2.9	329 (C_23_H_37_O)^−^	Unknown
20	64	—	371.2583	C_24_H_36_O_3_	3.0	327 (C_23_H_35_O)^−^	Unknown

RT: retention time; − indicates nonobserved/detected means.

**(a):** 

Microorganisms	HEG (mg/mL)		Gentamicin (*μ*g/mL)	
	MIC	MBC	MIC	MBC
*Gram-positive bacteria*				
*Staphylococcus aureus* ATCC 6538	5.16 ± 0.22	7.33 ± 0.16	1.66 ± 0.16	2.0 ± 0.28
Methicillin-resistant *S. aureus* ESA 175	5.75 ± 0.14	7.83 ± 0.16	1.83 ± 0.16	2.66 ± 0.16
Methicillin-resistant *S. aureus* ESA 159	6.25 ± 0.14	8.08 ± 0.22	2.0 ± 0.28	2.50 ± 0.28
*Enterococcus faecalis* ATCC 43300	6.75 ± 0.14	8.75 ± 0.25	2.16 ± 0.16	2.83 ± 0.30
Vancomycin-resistant *E. faecalis* ESA 201	7.33 ± 0.83	9.50 ± 0.28	2.33 ± 0.16	3.25 ± 0.14
Vancomycin-resistant *E. faecalis* ESA 361	7.58 ± 0.30	9.91 ± 0.54	2.66 ± 0.16	3.33 ± 0.16
*Gram-negative bacteria*				
*Escherichia coli* ATCC 29998	9.66 ± 0.44	13.0 ± 0.14	4.08 ± 0.08	4.58 ± 0.30
Cephalosporin-resistant *E. coli* ESA 37	10.25 ± 0.52	13.16 ± 0.44	4.66 ± 0.16	4.66 ± 0.22
Cephalosporin-resistant *E. coli* ESA 54	10.25 ± 0.25	13.33 ± 0.36	4.41 ± 0.08	4.91 ± 0.08
*Pseudomonas aeruginosa* ATCC 15442	11.91 ± 0.36	15.41 ± 0.36	4.75 ± 0.14	5.0 ± 0.28
Imipenem-resistant *P. aeruginosa* ESA 22	12.16 ± 0.60	15.41 ± 0.30	5.66 ± 0.16	6.61 ± 0.16
Imipenem-resistant *P. aeruginosa* ESA 23	12.75 ± 0.28	16.41 ± 0.36	6.66 ± 0.33	6.50 ± 0.28

**(b):** 

Microorganisms	HEG (mg/mL)		Amphotericin B (*μ*g/mL)	
	MIC	MFC	MIC	MFC
*Fungi*				
*Cryptococcus neoformans* ATCC 32264	18.08 ± 0.36	23.0 ± 0.14	0.55 ± 0.02	0.86 ± 0.07
Amphotericin B-resistant *C. neoformans* ESA 211	18.58 ± 0.71	24.08 ± 0.46	0.61 ± 0.06	1.25 ± 0.14
Amphotericin B-resistant *C. neoformans* ESA105	19.08 ± 0.82	24.25 ± 0.25	0.63 ± 0.01	1.66 ± 0.22
*Candida albicans* ATCC 10231	20.5 ± 0.28	28.25 ± 0.87	0.71 ± 0.04	0.91 ± 0.16
Amphotericin B-resistant *C. albicans* ESA 100	21.41 ± 0.54	29.33 ± 0.60	0.81 ± 0.04	1.66 ± 0.08
Amphotericin B-resistant *C. albicans* ESA 97	21.83 ± 0.44	31.08 ± 0.68	0.91 ± 0.01	1.75 ± 0.14
