# Supplementary material for: Chemical Composition and Pharmacological Effects of Geopropolis Produced by Melipona quadrifasciata anthidioides
Source: Oxid Med Cell Longev. 2017 Oct 26;2017:8320804. doi: 10.1155/2017/8320804 (PMC5682095; doi:10.1155/2017/8320804)
Supplement: Supplementary file 2 [file 8320804.f2.pdf]

Supplementary Material

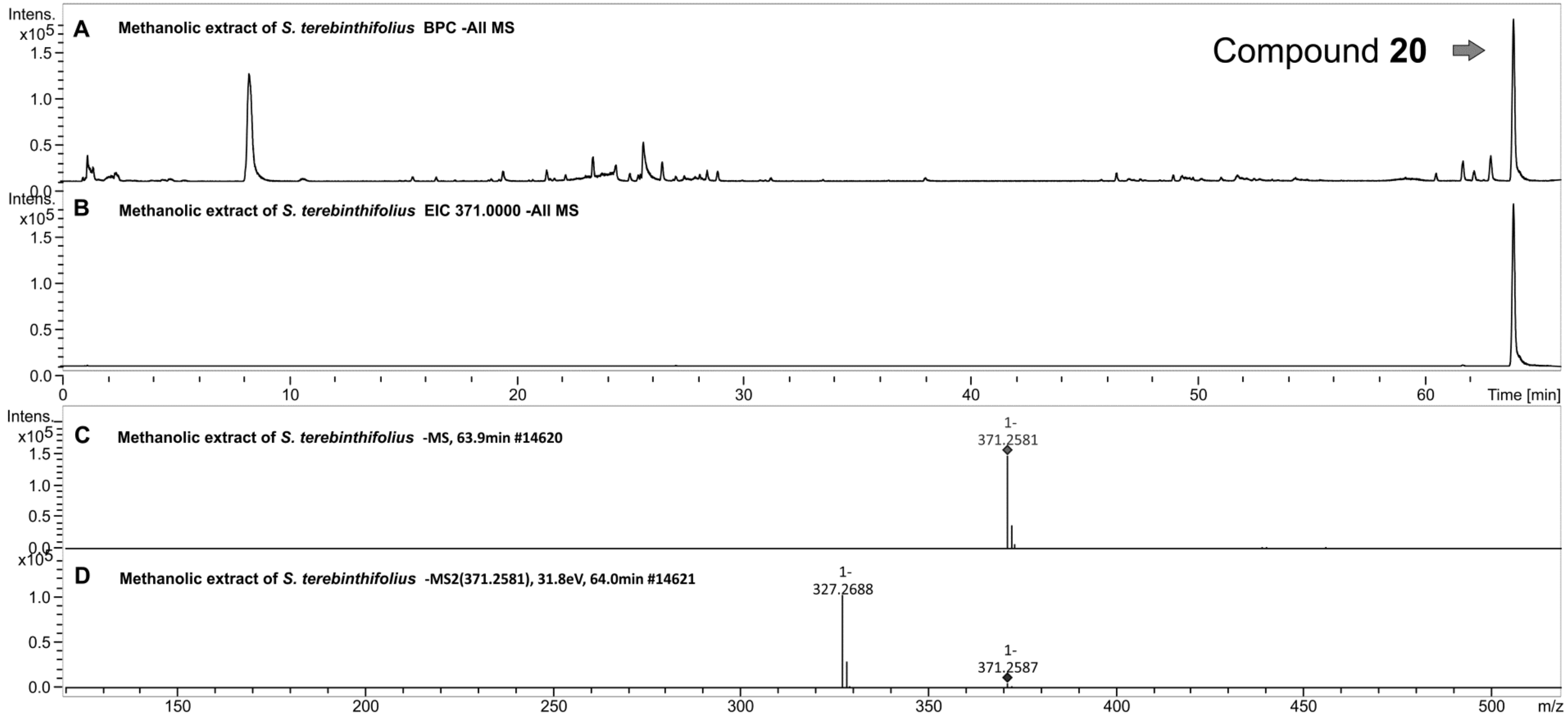

Supplementary Material. Base peak chromatogram of methanolic extract of the leaves of *Schinus terebinthifolius* in negative ionization mode.
